# Editorial: Chemosensitizing effect of natural products against cancers: Applications in enhancing chemotherapy and immunotherapy

**DOI:** 10.3389/fphar.2022.988226

**Published:** 2022-08-30

**Authors:** Nand K. Roy, Devesh Tewari, Maria Teresa Esposito

**Affiliations:** ^1^ Case Western Reserve University, Cleveland, OH, United States; ^2^ La Jolla Institute for Immunology, San Diego, CA, United States; ^3^ Department of Pharmacognosy and Phytochemistry, School of Pharmaceutical Sciences, Delhi Pharmaceutical Sciences and Research University, New Delhi, India; ^4^ School of Life and Health Science University of Roehampton, Roehampton, United Kingdom

**Keywords:** natural products, cancer, chemosensitization, chemotherapy, immunotherapy

Cancer is a complex phenomenon that encompasses over 200 different diseases that are the consequence of accumulation of genetic alterations (Roy et al., 2017). Despite the progress in the development of novel treatments for cancer, it still remains one of the prominent causes of death globally. Among the main reasons for the increasing rate of mortality is the emergence of chemoresistant or immunotolerant cancer cells that don’t respond to chemotherapy or immunotherapy modalities respectively (Maeda and Khatami 2018). The adverse events and toxicities associated with the available therapeutics can also pose a risk to patients. These challenges can be overcome by the utilization of natural products and their derivatives in the therapeutic regimen and it is well evident that many of the chemotherapeutic agents, currently used in the clinical setting, such as taxol, vincristine, irinotecan, etoposide, and paclitaxel etc. are derived from natural sources. Moreover, several researches have shown that the co-administration of natural products along with chemotherapeutic agents can increase their efficacy and even reverses chemoresistance. Therefore, this Research Topic was floated to attract researches and studies related to the potential of natural products in the chemosensitization of cancer cells to enhance the effect of chemotherapy and immunotherapy.

Interestingly, the single treatment of natural product such as tetramethylpyrazine (TMP) isolated from *Ligusticum chuanxiong* Hort is shown to be effective against different cancers at preclinical stages (Yang et al.). Another example of a such paradigm was discussed by Li et al., where they have reported the anticancer potential of gracillin, a steroidal saponin compound found in different medicinal plants such as *Rhizoma paridis*, *Pairs polyphylla*, *Dioscorea villosa*, *Acontum carmichaeli*, *Solanum incanum*, and *Solanum xanthocarpum* against non-small cell lung cancer (NSCLC) cells. Their research indicated that gracillin possesses anti-NSCLC activity through promoting autophagy of cancer that was regulated via the mTOR signaling pathway. Similarly, Chen et al. showed that α-hederin, a monodesmosidic triterpenoid saponin extracted from *Fructus akebiae* enhanced apoptosis while inhibiting the proliferation of hepatocellular carcinoma (HCC) cells through the alteration of Hippo/YAP protein signaling pathway. It was observed that the treatment of α-hederin treatment led to the increased level of proteins and genes associated with the Hippo signaling pathway. Moreover, their treatment also caused decreased nuclear YAP levels that consequently inhibited the proliferation while increasing the apoptosis of HCC cells.

Many researches over the past decades have suggested that the cancer cells develop resistance to the drugs and that can this can be circumvented by the combination of multiple drugs, acting on redundant signaling nodes. Such an effective combination approach has been studied in a case report of BRAFV600E-mutant colorectal cancer (CRC) (Cho et al.). A triple-regimen of vemurafenib that targets BRAFV600E, a topoisomerase I inhibitor (irinotecan), and cetuximab (an EGF-Receptor inhibitor, EGFRi) resulted in a complete response to the therapy. EGFRi can initiate many side effects and natural products in the therapeutic regimen can alleviate these challenges (Sui et al., 2020). In line with this, a randomized controlled study led by Liu et al. has shown that Honeysuckle, a natural product obtained from *Lonicera japonica* Thunb was effective in reducing acneiform rash incidences and severities induced by EGFRi such as cetuximab, erlotinib, gefitinib, and icotinib in colorectal and lung cancer patients. Moreover, the inclusion of natural bioactive components can also help in reversing the EGFRi resistance in cancer cells. Another study by Wu et al. highlighted the effect of Bruceine H, a derivative of *Brucea javanica* (L.), in overcoming resistance to receptor tyrosine kinase (RTK)-EGFRi in non small cell lung cancer (NSCLC) models. The authors have shown that the combination of Brucein H in the therapy increased the gefinitib response by suppressing Notch3, EGFR activation and β-catenin expression. Remarkably, the combination of Brucein H and gefinitib also induced Foxo3a, whose expression correlates with better response to EGFR inhibitors and better overall survival in NSLCC patients. Similar to this study, the comparative proteomic analysis by Cai et al. demonstrated the chemosensitizing effect of cryptotanshinone (CTS), a bioactive component of *Salvia miltiorrhiza* against gefitinib-resistant EGFR-mutant lung cancer cells. Lately, three proteins namely, catalase (CAT), heme oxygenase 1 (HMOX1), and stearoyl-CoA desaturase (SCD) identified through a proteomic analysis were validated as the important target of CTS and suggested to be a potential therapeutic target in lung cancers.

It is now well established that the cancer cells become resistant to certain kinds of therapy through the alterations of various signaling pathway (Mansoori et al., 2017). In accordance with this, Zheng et al. pointed out that the knockdown of Aldo-keto reductase 1C3 (AKR1C3) can sensitize the sorafenib-resistant hepatocellular carcinoma (HCC) cells through the inhibition of AKT kinase protein phosphorylation. Interestingly, the past literatures have indicated the importance of AKT kinase involvement in different types of cancers, and through *in silico* analysis it was also shown that it can potentially be targeted by natural products (Roy et al., 2020). In addition to targeting the Akt pathway by natural products, an interesting review by Fakhri et al. concluded that targeting the TLR/NF-κB/NLRP pathway with different kinds of bioactive phytocompounds such as phenolic compounds, alkaloids, terpenes/terpenoids, and sulfur compounds has the potential to overcome chemoresistance thereby can improve the outcome for chemotherapy and immunotherapy. Likewise, Dev et al.have discussed the role of curcumin, a polyphenol extracted from *Curcuma longa* as a RTK inhibitor. Curcumin possessed antitumorigenic effects on cancer cells through enhanced apoptosis and reduced cellular proliferation to reduced angiogenesis. These effects are mediated by inhibition of several signaling pathways including MAPK, PI3K/Akt, JAK/STAT, and NF-κB, which are often activated in response to treatment with single RTK inhibitors. An intriguing study by Yang et al. inferred that lumiflavin, a flavin analogue can diminish the cisplatin resistance of ovarian cancer stem-like cells (CSCs). Mechanistically, it was suggested that it induces its sensitization effect through the induction of inducing phenotypic differentiation of CSCs and it was due to the change in the notch signaling pathway and stem cell pathway.

Apart from natural resources derived by plants, recent years have witnessed a plethora of research that has emphasized the application of compounds derived by other natural sources or natural product-inspired synthesized molecule in cancer therapy (Newman and Cragg 2020). One such molecule is thymopentin (TP5), an immunomodulatory pentapeptide (49 amino acids) derived from the active fragment of a natural hormone called thymosin. Yu et al. have shown that TP5 can directly inhibit the stemness of colon cancer cells HCT116 as evident by reduced surface molecular markers associated with stemness such as CD133, CD44, and CD24 in addition to stemness-related genes like ALDH1, SOX2, Oct-4, and Nanog. These stemness-related changes thereby caused altered Wnt/β-catenin signaling that resulted in enhanced oxaliplatin (OXA) cytotoxicity against HCT116 cells. Also, Ragone et al.highlight the effectiveness of combination therapy of gemcitabine (Gem) and AdipoRon (AdipoR) in combating pancreatic ductal adenocarcinoma (PDAC) resistance to Gem. AdipoR is known to be the first synthetic orally active adiponectin receptor agonist suggested having an antitumorigenic effect against different types of cancers including PDAC. They have proposed that their combination halts the cell cycle progression in cancer cells effectively and p44/42 MAPK pathway involvement in the improved treatment outcomes.

Overall, the present Research Topic received a range of remarkable original and review articles that can improve the understanding of the chemosensitizing properties of natural products. Moreover, natural products other than plant sources such as biomolecules, toxins, venoms, ligands etc. obtained/inspired from microorganisms and animal sources also open new avenues for exploration to unravel nature’s treasure for drug discovery. However, the lack of articles in context to immunotherapy and clinical trials highlights the ample opportunity available in this area to fill the void in our understanding. Recent research by Messaoudene et al., 2022 have indicated the great potential of natural product in improving cancer immunotherapy and more research should be warranted in this field. Nevertheless, this Research Topic will be a great help to the scientific community to understand the relevance of natural products in cancer therapy and explore future possibilities and development in the area.
